# Proliferation Potential of 18-Month-Old Callus of *Ananas comosus* L. cv. Moris

**DOI:** 10.1100/tsw.2006.34

**Published:** 2006-02-17

**Authors:** A.E. De Silva, M.A. Kadir, M.A. Aziz, S. Kadzimin

**Affiliations:** ^1^ Department of Agricultural Biotechnology, Faculty of Agriculture, Universiti Putra Malaysia, 43400 Serdang, Selangor, Malaysia; ^2^ Department of Crop Science, Faculty of Agriculture, Universiti Putra Malaysia, 43400 Serdang, Selangor, Malaysia

**Keywords:** plant growth regulators, additives, pineapple

## Abstract

Differential effect of plant growth regulators and additives in proliferation of 18-month-old calli of *Ananas comosus* L. cv. Moris were assessed *in vitro*. The proliferation of callus relied on the growth regulators and additives. Of the different auxins supplemented in the Murashige and Skoog (MS) media, 32.22 μM α-naphthaleneacetic acid (NAA) gave the highest mean fresh weight of callus (46.817 g). Medium supplemented with 2,4-dichlorophenoxyacetic acid (2,4-D) was inferior to NAA, while b-naphthoxy acetic acid (BNOA) and p-chlorophenoxy acetic acid (4-CPA) were not effective in proliferating 18-months old callus. Addition of casein hydrolysate and coconut water to NAA supplemented medium showed better proliferation and production of callus. However, in terms of callus production, NAA at 32.22 μM was economically better.

## INTRODUCTION

Improvement of plants depends on variation and its retrieval, and subsequent regeneration as plantlets. Somaclonal variation, first emphasized by Larkin and Scowcroft[1], remains the simplest and easiest method for inducing variation. It is a useful tool for the improvement of crops. The potential of somaclonal variation in crops has been demonstrated in sugarcane[2], wheat[3], and sorghum[4], with highly stable variants that can be transmitted to progenies than other methods of induced mutations[4,5]. Callus, an unorganized mass of parenchyma cells of the outcome of mitosis, serves as a lead for the development of variants *in vitro*. Pineapple is a vegetatively propagating crop, and the improvement by conventional ways is tedious. Somaclonal variants with different characters have been reported in pineapple through the mediation of callus[6,7,8]. Besides the *in vitro* culture conditions, the age of the callus plays a significant role in the frequency of variants, and an enhanced variability with age has been documented in regenerated banana plants (*Musa* spp.)[9], suspension cultures of rice calli (*Oryza sativa* L.)[10,11], and garlic (*Allium sativum* L.)[12]. Thus, an optimization of media after specific periods in order to retain the totipotency of cells for longer period (the most crucial factor in somaclonal variation) is mandatory for the continuous maintenance of the callus. The study focused on the continuous maintenance and proliferation potential of 18-month-old callus of pineapple cv. Moris in relation to growth regulators and additives with the aim of developing high-frequency somaclonal variants.

## MATERIALS AND METHODS

One gram callus of pineapple cv. Moris (friable, light green, and yellowish) was used as inoculum. It has been grown on MS medium with 42.96 µ*M* NAA for 18 months. The basal medium used in this study was MS media [5] supplemented with 3% (w/v) sucrose and solidified with 0.4% (w/v) Gelrite™. The pH of the medium was adjusted to 5.8 prior to the addition of gelling agent. The basal medium was modified with the addition of auxins [α-naphthaleneacetic acid (NAA), 2,4-dichlorophenoxyacetic acid (2,4-D), b-naphthoxy acetic acid (BNOA), and p-chlorophenoxy acetic acid (4-CPA)] and additives [casein hydrolysate (CH) and coconut water (CW)] alone or in combinations. The study consisted of two phases. Phase 1 assessed the potential of various auxins on callus proliferation and phase 2 assessed the potential of media with different levels of CH and CW supplemented to MS medium containing 32.22 µ*M* NAA (optimal medium in the first phase).

In the first phase, MS medium was supplemented with various levels of NAA (10.74, 21.48, 32.22, 42.96, 53.71, and 64.44 µ*M*), 2,4-D (2.26, 4.52, 9.05, 18.10, 27.14, and 36.19 µ*M*), BNOA (2.47, 9.89, 19.70, 29.67, 39.56, and 49.45 µ*M*), and 4-CPA (2.68, 10.72, 21.44, 32.16, 42.87, and 53.59 µ*M*) (Table 1). The levels tested for NAA and 2,4-D were selected based on a preliminary study performed in our laboratory. Auxins, 4-CPA and BNOA were selected because of their low price. The costs of 25 g of each auxin were as follows: NAA = USD 15.20, 2,4-D = USD 4.45, 4-CPA = USD 20.20, and BNOA = USD 12.60[13]. In the second phase, different levels of CH (0, 100, 200, 300, and 400 mg/l) and CW (0, 5, 10, 15% v/v) were supplemented to MS medium containing 32.22 µ*M* NAA either singly or in combination (Table 2). The callus used in the second phase was from the 18-month-old batch (now 21 months old). In the present study, MS basal medium and MS medium with 32.22 µ*M* NAA served as control 1 and control 2, respectively. The production costs of callus with respect to the prices of growth regulators and additives were also assessed.

The cultures were subcultured at an interval of 4 weeks for 12 weeks. All the cultures were incubated at 25 ± 2°C with 16-h light (at an irradiance of 37.5 µmol $m^{-2}$ $s^{-1}$)/8-h light cycle, and relative humidity of 50–60%. After the removal of gelling agents and blot drying with sterile filter paper, the fresh weight of the calli (including inoculum weight) were recorded at the end of 12 weeks.

A total of 25 treatments (including a control, MS hormone-free media) were studied. The treatments were replicated 12 times in a Randomized Complete Block Design. Analysis of Variance (ANOVA) was used to test for treatments effect and Duncan's Multiple Range Test (DMRT) at $p \leq 0.05$ was used to compare treatment means. The statistical software used was the Statistical Analysis System (SAS)[14].

## RESULTS AND DISCUSSION

### Effects of Auxins

The calli (18 months old) cultured on NAA treatments yielded the highest mean fresh weight (Table 1) and were superior in all aspects to those grown on media with other auxins. The callus produced on NAA-supplemented medium showed similar features of the inoculum such as friable and light green-yellow color (Fig. 1) as compared to auxins 2,4-D, BNOA, and 4-CPA, and control, which showed occasional calli browning and hard globular meristemic bodies (which were responsible for the formation of shoots from callus on these media). Generally, media supplemented with 2,4-D, BNOA, and 4-CPA did not favor calli proliferation as they were slow and calli gradually became dark brown. However, hard meristemic globular bodies formed from existing calli led to the formation of some shoots.
The effectiveness of NAA in the proliferation of pineapple callus was consistent with earlier reports[6,15].

**TABLE 1**
**Effect of Different Auxins in Proliferation of 18-Month-Old Callus**

| Plant Growth Regulators (μ*M*) | | | | Mean Fresh Weight of Callus (g) |
|---|---|---|---|---|
| NAA | 2,4-D | BNOA | 4-CPA | |
| 00 (Control 1) | | | | 1.597$^{g}$ |
| 10.74 | | | | 21.921$^{bc}$ |
| 21.48 | | | | 26.652$^{ab}$ |
| 32.22 | | | | 36.754$^{a}$ |
| 42.96 | | | | 26.683$^{ab}$ |
| 53.71 | | | | 30.927$^{a}$ |
| 64.44 | | | | 29.247$^{ab}$ |
| 00 | 2.26 | | | 2.803$^{fg}$ |
| 00 | 4.52 | | | 5.995$^{efg}$ |
| 00 | 9.05 | | | 4.579$^{fg}$ |
| 00 | 18.10 | | | 14.682$^{cde}$ |
| 00 | 27.14 | | | 21.145$^{bc}$ |
| 00 | 36.19 | | | 15.933$^{cd}$ |
| 00 | 00 | 2.47 | | 1.982$^{fg}$ |
| 00 | 00 | 9.89 | | 3.729$^{fg}$ |
| 00 | 00 | 19.70 | | 2.848$^{fg}$ |
| 00 | 00 | 29.67 | | 6.021$^{efg}$ |
| 00 | 00 | 39.56 | | 6.653$^{efg}$ |
| 00 | 00 | 49.45 | | 4.643$^{fg}$ |
| 00 | 00 | 00 | 2.68 | 3.799$^{fg}$ |
| 00 | 00 | 00 | 10.72 | 6.107$^{efg}$ |
| 00 | 00 | 00 | 21.44 | 10.685$^{defg}$ |
| 00 | 00 | 00 | 32.16 | 10.242$^{defg}$ |
| 00 | 00 | 00 | 42.87 | 11.441$^{def}$ |
| 00 | 00 | 00 | 53.59 | 11.441$^{def}$ |

Means followed by the same letter are not significantly different at $p \leq 0.05$.

Control 1 = MS alone.

Data represent mean value of 12 replicates. Mean callus fresh weight includes inoculum weight. Incubation period was 12 weeks.

Increase in NAA level did significantly improve mean fresh weight of calli (Table 1). Nevertheless, the mean fresh weight of 53.71 μ*M* was not significantly different from that of NAA at 32.22 μ*M* (Table 1). The medium with 32.22 μ*M* NAA was considered the best treatment for high calli proliferation and was used in the second phase as control 2 of this phase.

The inoculum used in this study was 18-month-old callus maintained on 42.96 μ*M* NAA. The mean fresh weight of the callus on 21.48 μ*M* NAA was not significantly different from that of 42.96 μ*M* NAA (Table 1). The proliferation of callus at lower levels of NAA after 18-month maintenance on the medium with higher levels of NAA indicates the acquisition of endogenous and exogenous balance of the growth regulators favoring the proliferation of the 18-month-old callus[16]. At higher levels of NAA (53.71 and 64.44 μ*M*), the mean fresh weight of their calli were not significantly different from 42.96 μ*M* NAA.

**TABLE 2**
**Effect of Additives in Proliferation of 21-Month-Old Callus**

| Additives | | Mean Fresh Weight of Callus (g) |
|---|---|---|
| CH (mg/l) | CW (%) | |
| Control 1 | | 1.7567[d] |
| Control 2 | | 46.817[c] |
| 100 | 0 | 56.470[bc] |
| 200 | 0 | 55.820[bc] |
| 300 | 0 | 53.093[bc] |
| 400 | 0 | 58.931[b] |
| 00 | 5 | 53.142[bc] |
| 00 | 10 | 57.040[bc] |
| 00 | 15 | 56.507[bc] |
| 100 | 5 | 57.253[b] |
| 100 | 10 | 53.462[bc] |
| 100 | 15 | 55.239[bc] |
| 200 | 5 | 58.300[b] |
| 200 | 10 | 60.319[b] |
| 200 | 15 | 58.539[b] |
| 300 | 5 | 56.166[bc] |
| 300 | 10 | 58.298[b] |
| 300 | 15 | 66.586[a] |
| 400 | 5 | 55.522[bc] |
| 400 | 10 | 57.729[b] |
| 400 | 15 | 58.948[b] |

Means followed by the same letter are not significantly different at $p \leq 0.05$.

All media except Control 1 contained 32.22 μ*M* NAA.

Control 1 = MS alone.

Control 2 = MS + 32.22 μ*M* NAA (treatment that gave highest calli fresh weight in Study 1).

Data represent mean value of 12 replicates. Mean callus fresh weight includes inoculum weight. Incubation period was 12 weeks.

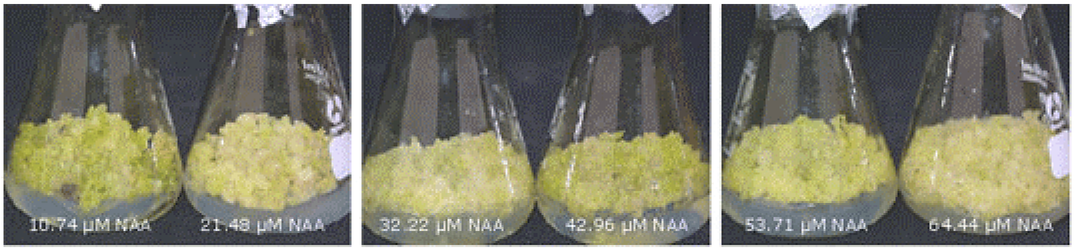

**FIGURE 1**. Calli proliferation of Moris cultivar under six different levels of NAA, after 12 weeks of culture.

The efficacy of NAA in callus proliferation was followed by 2,4-D (Table 1). This observation was consistent with the report of Matthews and Rangan[6]. However, calli on lower levels of 2,4-D (2.26, 4.52, and 9.05 μ*M*) showed brown patches of calli similar to that observed in Control 1, but with fewer regenerated shoots (Fig. 2). According to de Klerk et al., the rate of uptake of auxins is different. NAA is six times faster than indole-3-acetic acid[17]. The mobilization of 2,4-D in tissues has been considered to be slow[18]. The difference in the uptake and metabolism of auxins may also be a reason for the differential response of NAA and 2,4-D.

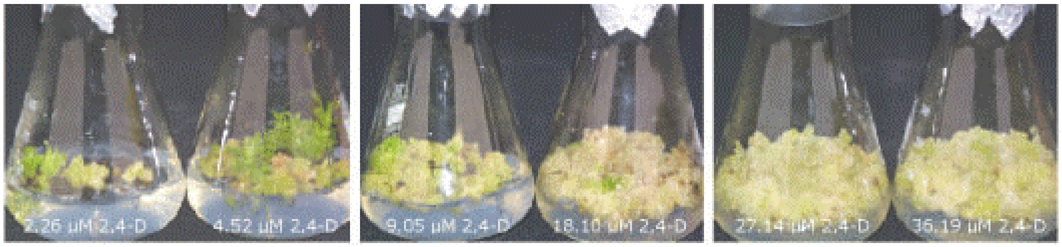

**FIGURE 2.** Calli proliferation of Moris cultivar under six different levels of 2,4-D, after 12 weeks of culture.

Calli cultured on medium with BNOA and 4-CPA were inferior in terms of proliferation to NAA- and 2,4-D–supplemented media (Table 1). Except for the higher levels of 4-CPA (42.87 and 53.59 μ*M*), which were significantly different from control, no significant differences were observed among the other treatments and control (Table 1). Similar to control, brown patches of calli were also noticed on the calli produced on BNOA and 4-CPA.

## Effect of Additives

The different levels of CH and CW either alone or in combination with 32.22 μ*M* NAA exhibited differential rate of proliferation of callus (Table 2). The calli in all cases were yellowish to green in color except for Control 1 (Fig. 3). Synergistic effect of auxins with CW or CH alone or both has already been accomplished in pineapple[6].

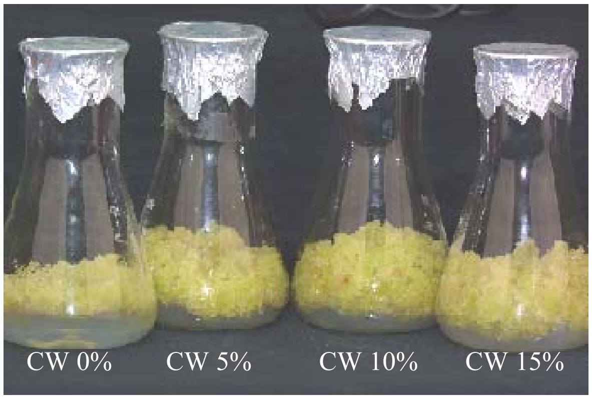

**FIGURE 3.** Calli proliferation under CH 300 mg/L at different CW levels, after 12 weeks of culture.

MS medium containing 32.22 μ*M* NAA supplemented with 300 mg/l CH and 15% CW yielded the highest mean calli fresh weight. However, the proliferation and maintenance of calli on MS medium having

53.71 μ*M* NAA with 400 mg/l CH and 15% CW has been demonstrated in another commercial pineapple cultivar[6]. The difference may be due to the difference in medium supplements.

Assessment of callus yield per USD clearly showed that MS medium with 32.22 μ*M* NAA alone was most economical for the production of callus in pineapple cv. Morris (Table 3) in spite of the higher biomass of callus of some treatments with NAA and CW or CH or both.

**TABLE 3**
**Production of Callus (Fresh Weight) of cv. Moris per USD as to Different Treatments**

| CW (%):CH (mg/L) | Cost of Treatment (USD) per Liter Medium (CW + CH + 32.22 μ*M* NAA = Total) | Treatment Cost Inclusive of Two Subcultures (USD) | Mean Calli Fresh Weight (g) | 1 USD:Mean Calli Fresh Weight (g) |
|---|---|---|---|---|
| 15:300 | 0.0080 + 0.0421 + 0.0036 = 0.0537 | 0.1611 | 66.586 | 413.32 |
| 10:200 | 0.0053 + 0.0280 + 0.0036 = 0.0369 | 0.1107 | 60.319 | 544.89 |
| 15:400 | 0.0080 + 0.0560 + 0.0036 = 0.0676 | 0.2028 | 58.948 | 290.67 |
| 0:400 | 0.0000 + 0.0560 + 0.0036 = 0.0596 | 0.1788 | 58.931 | 329.59 |
| 15:200 | 0.0080 + 0.0280 + 0.0036 = 0.0396 | 0.1188 | 58.539 | 492.75 |
| 5:200 | 0.0027 + 0.0280 + 0.0036 = 0.0314 | 0.0948 | 58.300 | 618.90 |
| 10:300 | 0.0053 + 0.0421 + 0.0036 = 0.0510 | 0.1530 | 58.298 | 381.03 |
| 10:400 | 0.0053 + 0.0560 + 0.0036 = 0.0649 | 0.1947 | 57.729 | 296.50 |
| 5:100 | 0.0027 + 0.0140 + 0.0036 = 0.0203 | 0.0609 | 57.253 | 935.51 |
| 10:0 | 0.0053 + 0.0421 + 0.0036 = 0.0510 | 0.1530 | 57.040 | 372.81 |
| Control 2 | 0.0036 | 0.0108 | 46.817 | 4334.91 |

Control 2 = MS + 32.22 μ*M* NAA (treatment that gave highest calli fresh weight in Study 1).

Price of NAA = USD 15.20/25 g (Sigma-Aldrich, 2004/2005)
= USD 0.0006/mg

Price of CH = USD 35.10/250 g (Sigma-Aldrich, 2004/2005)
= USD 0.00014/mg

Price of CW = USD 1 for 1.9 l (Local market)
= USD 0.00053/ml

Data represent mean of callus fresh weight produced per USD for selected treatments.

## CONCLUSIONS

Addition of CH and CW to NAA-supplemented medium showed better proliferation and production of callus. However, in terms of callus production, NAA at 32.22 μ*M* was economically better.

## BIOSKETCH

**A.E. De Silva** is currently a research assistant at Monash University Malaysia and has a Masters degree in Agricultural Biotechnology. Her areas of research interests are genetic engineering of agricultural crops mainly through *Agrobacterium*-mediated transformation, gene expression; and plant tissue culture techniques such as micropropagation, somaclonal variation, organogenesis, and secondary metabolite production.
